# Latest Nanoparticles to Modulate Hypoxic Microenvironment in Photodynamic Therapy of Cervical Cancer: A Review of In Vivo Studies

**DOI:** 10.3390/ijms26178503

**Published:** 2025-09-01

**Authors:** Dorota Bartusik-Aebisher, Mohammad A. Saad, Agnieszka Przygórzewska, Paweł Woźnicki, David Aebisher

**Affiliations:** 1 Department of Biochemistry and General Chemistry, Collegium Medicum, Faculty of Medicine, University of Rzeszów, 35-310 Rzeszów, Poland; dbartusikaebisher@ur.edu.pl; 2 Wellman Center for Photomedicine, Massachusetts General Hospital and Harvard Medical School, Boston, MA 02114, USA; msaad1@mgh.harvard.edu; 3 English Division Students Science Club, Collegium Medicum, Faculty of Medicine, University of Rzeszów, 35-310 Rzeszów, Poland; ap117623@stud.ur.edu.pl; 4 Doctoral School, Collegium Medicum of the University of Rzeszów, 35-310 Rzeszów, Poland; pawelw@dokt.ur.edu.pl; 5 Department of Photomedicine and Physical Chemistry, Collegium Medicum, Faculty of Medicine, University of Rzeszów, 35-310 Rzeszów, Poland

**Keywords:** PDT, cervical cancer, hypoxia, cancer

## Abstract

Photodynamic therapy (PDT) is a promising, minimally invasive treatment for cervical cancer, but its efficacy is significantly limited by hypoxia—oxygen deficiency in the tumour microenvironment. The aim of this study was to present strategies to counteract hypoxia in PDT using the latest nanotechnologies. Based on a review of the literature available in PubMed/MEDLINE, Scopus, and Web of Science databases, covering the period from January 2024 to March 2025, nine original in vivo studies were identified that investigated the use of nanoparticle-based strategies to overcome hypoxia and enhance the efficacy of PDT in cervical cancer. A variety of approaches to improve tumour oxygenation are described, including the catalytic decomposition of hydrogen peroxide (H_2_O_2_) with manganese oxide (MnO_2_), the use of bimetallic nanozymes (e.g., Au_2_Pt), and FeOOH structures and oxygen storage and control systems (e.g., endoperoxides). Strategies to reduce oxygen consumption by cancer cells, such as nitric oxide (NO) release or inhibition of mitochondrial oxidative phosphorylation, are also discussed. The review shows that appropriately designed nanoparticles can effectively counteract hypoxia, enhancing the efficacy of PDT by intensifying reactive oxygen species (ROS) generation and modulating HIF-1α factor expression. The strategies presented here have the potential to significantly improve the efficacy of photodynamic therapy in the treatment of cervical cancer, especially under conditions of limited oxygen availability.

## 1. Introduction

Cervical cancer represents the fourth most common malignancy in women worldwide, second only to breast cancer, colorectal cancer, and lung cancer. Globally, there were approximately 570,000 new cases and 311,000 deaths from this cancer in 2018. The age-standardised incidence rate at the time was 13.1 per 100,000 women [1,2]. Cervical cancer is also the most commonly diagnosed malignancy in pregnancy, with an incidence estimated at 0.1–12 cases per 10,000 pregnancies. The median age at diagnosis is now 55 years and has decreased by about 15 years over the past two decades. Approximately 25 per cent of cases are in women under 35 years of age [3]. The pathogenesis of cervical cancer is multifactorial. Well-documented risk factors include smoking, early initiation of sexual intercourse, sexual multiparity, long-term use of oral contraceptives, low socioeconomic status, and immunosuppression related to HIV infection or iatrogenic immunosuppression. However, a key role in the development of this cancer is played by chronic infection with oncogenic human papilloma virus (HPV) types [4,5,6,7]. Approximately 80% of all cases of cervical cancer are squamous cell carcinoma. In recent decades, however, there has been an increase in the proportion of cervical adenocarcinoma, which was previously much rarer. Other less common histological subtypes include adeno-epithelial, serous papillary, and neuroendocrine carcinoma [8,9].

The primary factor determining the choice of therapeutic strategy is the clinical stage of the tumour, usually assessed according to the classification of the International Federation of Gynaecology and Obstetrics (FIGO). Also of importance are the histological type, the patient’s age, the presence of comorbidities, and, if possible, the preference for fertility preservation [10,11,12]. In the early, micro-invasive stages (IA1-IA2), the recommended treatment is conisation or trachelectomy in women planning to become mothers, while in other cases, simple hysterectomy is the recommended treatment. In the absence of consent to surgical treatment, neoadjuvant radiotherapy with further follow-up is an option. In stages IB1-IIA1, the treatment of choice is radical hysterectomy with pelvic lymphadenectomy, usually supplemented with chemoradiotherapy. In advanced stages (IIB-IVA), combined chemoradiotherapy remains the standard treatment. Surgical treatment in these cases can only be considered in individually qualified cases where radical resection can be achieved. Otherwise, surgery may lead to an unfavourable delay of systemic therapy and be associated with significant surgical risks in patients in poorer general condition. The 5-year survival rate for advanced/metastatic cervical cancer is low, ~15–30% which highlights the need for alternative therapies [13,14,15,16,17,18].

Photodynamic therapy (PDT) is a minimally invasive, selective method of treating cancer using the interaction of three key elements: light at a specific wavelength, a photosensitiser (PS), and molecular oxygen (O_2_) [19,20,21,22]. The mechanism of action of PDT is through excitation of a PS by exposure to light, which leads to its transition from the ground state to the triplet state. In this state, the PS can participate in two types of photochemical reactions: type I, involving the transfer of electrons or protons and the generation of radicals, and type II, in which energy is transferred directly to the oxygen molecule in the ground state (^3^O_2_), leading to the formation of reactive singlet oxygen (^1^O_2_) [23,24,25]. Of these mechanisms, the predominant pathway for the generation of cytotoxic reactive oxygen species in an anticancer context is the type II reaction. The reactive oxygen species generated induce cell death by apoptosis, necrosis, or autophagy, damage tumour vascular structures, and can activate the host immune response [26,27]. Although PDT offers significant advantages, including precise local action, low systemic toxicity, and no cumulative side effects, its clinical efficacy remains limited by its dependence on the availability of molecular oxygen, particularly in solid tumours [28,29].

One of the most serious limitations of PDT is the presence of hypoxia within the tumour, a state of oxygen deficiency that results from inefficient and chaotic vascularisation and a rapid rate of tumour cell proliferation [30,31]. The oxygen partial pressure in hypoxic tissues usually falls below 2.5 mm Hg, whereas in healthy tissues these values exceed 40 mm Hg, allowing metabolic processes to function properly [32,33]. Tumour hypoxia promotes tumour progression, invasiveness, angiogenesis, and increases the risk of resistance to treatment [34,35,36,37,38,39].

High oxygen consumption during PDT treatment can lead to a paradoxical effect, the further aggravation of local hypoxia [40]. As a result of the reduction in oxygen supply and its intensive consumption during photodynamic reactions, the amount of reactive oxygen species generated decreases over successive therapy cycles, significantly reducing their effectiveness [41,42,43,44,45]. At the same time, hypoxia promotes cancer cell adaptation, leading to changes in gene expression, proteomics, and cell phenotype that promote cell survival under stress conditions [46,47]. This is particularly the case for decreased susceptibility of cells to apoptosis, increased resistance to anticancer agents, and increased development of tumourigenic features [48]. At the molecular level, the main regulator of the cellular response to hypoxia is hypoxia-inducible factor 1 (HIF-1). HIF-1α, stabilised under conditions of low oxygen partial pressure, activates a number of genes responsible for metabolic adaptation, angiogenesis, and drug resistance. Of particular importance is the MDR1 gene, which encodes P-glycoprotein, an ABC family transport protein responsible for actively removing cytostatic drugs from the cell, leading to a reduction in their intracellular concentration [49,50]. In addition, HIF-1 induces a number of autophagy-related proteins, such as BNIP3, Beclin-1, ATG5, ATG7, and ATG9A, thereby promoting tumour cell survival under conditions of oxidative stress and nutrient deprivation [51,52,53,54,55]. A key role in this process is also played by inhibition of the activity of the mTORC1 complex, a central regulator of cell growth, which, under conditions of hypoxia and nutrient limitation, initiates the autophagy pathway [56,57]. Worsening hypoxia during PDT can also contribute to the closure of local blood vessels, which further restricts oxygen supply to the tumour and reduces the effectiveness of the therapy [51]. In practice, this means that after several episodes of PDT, the amount of reactive oxygen species generated decreases dramatically, and the cancer cells are given time to adapt and develop resistance [58,59].

Hypoxia is, therefore, one of the main limitations to the effectiveness of PDT. In recent years, there has been increased interest in the use of nanotechnologies to modulate the tumour microenvironment, including improved oxygenation of tumour tissues [26]. Nanoparticles (NPs), defined as structures with at least one dimension between 1 and 100 nm [60,61,62], provide a promising platform for the design of advanced drug delivery systems. Among the strategies under development, nanoparticles designed to actively counteract hypoxia by catalytically generating oxygen in situ by controlled release of stored singlet oxygen and by reducing oxygen consumption by cancer cells are of particular interest. These systems not only enable local tumour reoxidation but often integrate different mechanisms of action, offering high selectivity, the possibility of precise drug release, and synergistic enhancement of the effect of photodynamic therapy.

Our review summarises research on the utility of nanoparticles in overcoming hypoxia during cervical cancer targeted PDT, published between 2024 and March 2025. An article search was conducted in the PubMed/MEDLINE, Scopus, and Web of Science databases on 17 March 2025, using the phrase “PDT AND nanoparticles”. A total of 2063 articles were identified. The inclusion and exclusion criteria of the retrieved articles for this review are presented in Table 1. Finally, nine original scientific articles describing nanoparticles in photodynamic therapy for cervical cancer performed in vivo to overcome hypoxia were eligible for the study.

## 2. H_2_O_2_ Decomposition

One promising strategy for increasing oxygen availability in areas of tumour hypoxia is the use of targeted compounds capable of converting endogenous hydrogen peroxide (H_2_O_2_) into O_2_ directly inside the tumour. Tumour cells exhibit significantly higher concentrations of H_2_O_2_ than normal cells, creating a selective advantage for this approach [63]. Furthermore, a decrease in H_2_O_2_ levels in cancer cells can lead to inhibition of proliferative pathways [64], including those regulated by HIF-1α and NF-κB [65,66], which may further enhance the anti-tumour effect. Three approaches have been used in nanoparticles dedicated to PDT of cervical cancer: the use of manganese (II) oxide (MnO_2_), Au_2_Pt nanozyme, and FeOOH.

### 2.1. Manganese (II) Oxide

**Figure 1:**
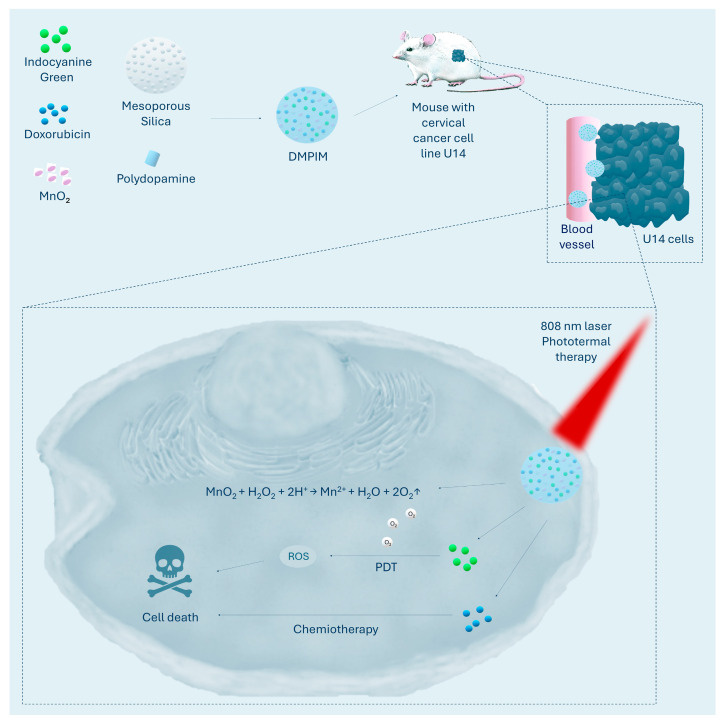
The figure shows DMPIM nanoparticles composed of mesoporous silica, doxorubicin, polydopamine, indocyanine green, and MnO_2_. After tumour accumulation, MnO_2_ generates oxygen to enhance photodynamic therapy, 808 nm laser irradiation activates indocyanine to produce reactive oxygen species, polydopamine and indocyanine induce photothermal effects, and doxorubicin release provides complementary chemotherapy. O_2_↑ indicates an increase in oxygen levels [68].

MnO_2_ can effectively degrade intracellular H_2_O_2_ in the acidic tumour microenvironment to produce oxygen and alleviate hypoxia during PDT [67]. A MnO_2_-based strategy to combat hypoxia was applied in the studies reported by Yang et al. [68], who developed a DMPIM nanoparticle composed of mesoporous silica loaded with MnO_2_, the photosensitiser indocyanine green, and doxorubicin, all coated with a layer of polydopamine. It was confirmed that the MnO_2_ layer in the DMPIM nanoparticles reacts with H_2_O_2_, generating O_2_ and consequently increasing ROS production under irradiation relative to the nanoparticles without MnO_2_, demonstrating the key role of MnO_2_ in enhancing the photodynamic effect. Incorporation of MnO_2_ into the nanoparticles contributed to a significant reduction in HeLa cell survival in vitro compared to nanoparticles lacking this component, and showed the highest efficacy in inhibiting tumour growth in a mouse model of U14 tumours, achieving 92.91% inhibition versus 88.25% in the group without MnO_2_. Figure 1 shows the mechanism of the MnO_2_ reaction and the structure and mode of action of DMPIM nanoparticles [68]. Cai et al. [69] developed a DPIMGC nanoparticle composed of a core containing doxorubicin surrounded by polydopamine, a layer of indocyanine green, a shell of MnO_2_, and an outer layer of celecoxib-conjugated gelatin. Manganese dioxide-functionalised nanoparticles (MnO_2_-NPs) showed significantly increased generation of reactive oxygen species compared to non-functionalised nanoparticles, which translated into higher cytotoxicity against the HeLa cell line in vitro. In a preclinical cervical cancer model, developed by the implantation of U14 cells in the axilla of mice, MnO_2_-NPs led to the strongest inhibition of tumour growth, achieving an inhibition rate of 91.47%. Furthermore, tumour tissues from MnO_2_-NPs-treated animals showed a marked reduction in HIF-1α factor expression, suggesting an effective reduction of hypoxia in the tumour microenvironment. In comparison, the group receiving MnO_2_-free NPs showed a lower level of tumour growth inhibition (77.06% inhibition) and HIF-1α levels similar to those observed in the control group [69]. Wang et al. [70] designed the M-HMnO_2_@ICG nanoplatform, which consists of a hollow mesoporous MnO_2_ core that acts as both carrier and catalyst due to its large specific surface area. The poly (allylamine hydrochloride) coating allows efficient loading of indocyanine green, while being surrounded by a cell membrane derived from the HeLa line ensures selective binding to cancer cells of the same phenotype and avoidance of phagocytosis. The HMnO_2_ core plays a key role as a multifunctional catalytic component, showing synergistic effects in cancer therapy. Firstly, it catalyses the decomposition of hydrogen peroxide to molecular oxygen, helping to improve oxygenation in the tumour microenvironment. Secondly, it increases the efficiency of the conversion of near-infrared (NIR) light into heat, which intensifies the photothermal effect. Thirdly, by releasing Mn^2+^ ions, it initiates the Fenton reaction, generating hydroxyl radicals, enabling chemodynamic therapy. Importantly, HMnO_2_ degrades selectively in the acidic and reducing tumour microenvironment, leading to the controlled release of both ICG and Mn^2+^ ions. In vivo studies conducted on a mouse model of HeLa tumours showed a significant reduction in HIF-1α factor expression in groups treated with M-HMnO_2_@ICG in combination with NIR irradiation, indicating an effective reoxidation of the tumour environment induced by the nanoparticles. Furthermore, the M-HMnO_2_@ICG + NIR treatment resulted in 88% inhibition of tumour growth in a HeLa mouse model, confirming its high anti-tumour efficacy [70].

### 2.2. Metallic Nanozymes

Metallic nanozymes are another type of artificial catalase [71]. The catalytic activity of single noble metals is often limited by the tumour microenvironment. Bimetallic nanozymes using the synergistic effect of two atoms are the answer to this problem. They feature both superior catalytic and multi-enzymatic properties compared to single metal nanozymes [72]. Bimetallic Au_2_Pt nanozymes were used by Liu et al. [73]. These nanozymes exhibit properties similar to both catalase and peroxidase [74]. The LRZAPH (LuAG:Tb/Ce-RB@ZIF-8-Au_2_Pt-HA) nanoplatform designed by Liu et al. consists of a core of scintillating LuAG nanoparticles doped with terbium and cerium ions and a Rose Bengal photosensitiser. The core is surrounded by a pH-sensitive ZIF-8 coating, which protects the structure and allows selective release of the components in the acidic tumour microenvironment. Au_2_Pt nanozymes are embedded in the coating. The entire nanoplatform is coated with a layer of hyaluronic acid. Studies have shown that Au_2_Pt exhibits catalase activity over a wide pH range from weakly acidic to basic, with maximum efficiency at pH ≈ 8, while under low pH conditions, peroxidase-like reactions leading to the generation of -OH radicals dominate, at the expense of catalase activity [73]. This is crucial because hyaluronic acid-functionalised nanoparticles accumulate in endosomes and lysosomes [75], where the pH is acidic [76]. Compared to the control group (LH; LuAG:Tb/Ce-RB), in in vitro studies on HeLa cells, LRZAPH exhibited significantly higher generation of reactive oxygen species, greater cytotoxicity, and more effective long-term inhibition of colony formation (colony survival rate: LRZAPH ~20% vs. LH ~40%). In a mouse model of HeLa tumours, LRZAPH therapy after X-ray irradiation led to 93% inhibition of tumour growth. It should be noted, however, that the control nanoparticles were devoid not only of Au_2_Pt, but also ZIF-8 and HA groups [73]. Therefore, the observed differences may be due to both Au_2_Pt activity and pH-dependent component release and targeted delivery via hyaluronate, and it is not possible to determine the exact role of Au_2_Pt in the efficacy of LRZAPH.

### 2.3. FeOOH

**Figure 2:**
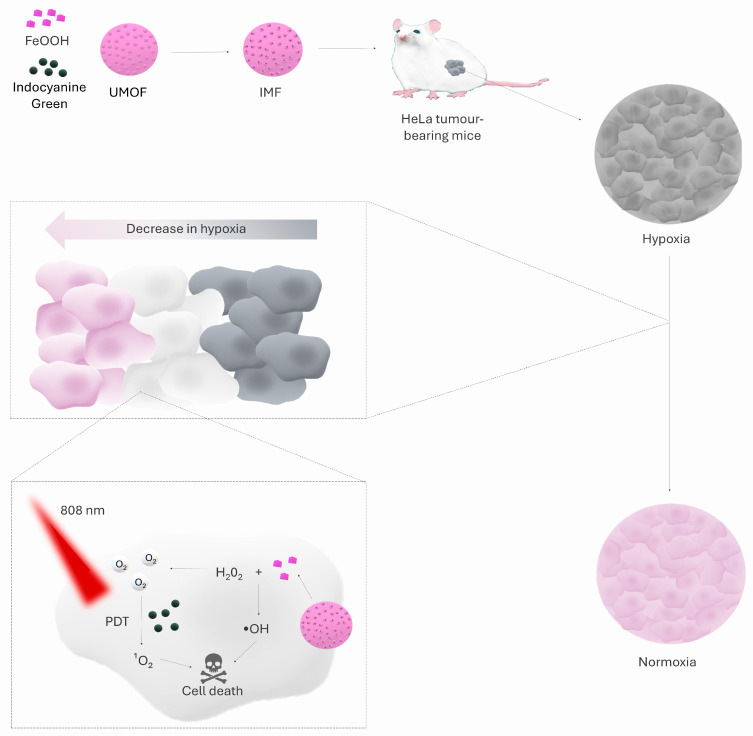
The figure shows IMF nanoparticles, composed of UMOF, FeOOH, and ICG. In hypoxic tumours, FeOOH converts H_2_O_2_ into O_2_ and cytotoxic •OH, which, together with ICG-activated singlet oxygen under 808 nm light, drive a synergistic photodynamic therapy effect. Arrow shows direction from higher to lower intensivity of hypoxia in tumor [78].

Coordinated iron ions exhibit artificial enzymatic activity [77] and can function in a similar manner to catalase. This property was exploited by Gao et al. [78] by synthesising an IMF nanoparticle (ICG ⊂ UMOF@FeOOH). Its structural core is a modified UiO-66-(COOH)_2_ metal-organic framework (UMOF), developed from a hexameric Zr_6_O_4_(OH)_4_(CO_2_)_12_ cluster linked by a 1,2,4,5-benzenetetetracarboxylic ligand (H_4_btec). A thin amorphous FeOOH film was deposited on the UMOF surface, acting as catalase-like active centres, due to the strong coordination of Fe^3+^ ions with -COOH groups. Indocyanine green was loaded into the UMOF pore. In vitro studies on HeLa cells showed that the IMF nanoparticle generated molecular oxygen under both normoxia (21% O_2_) and hypoxia (2% O_2_) conditions, translating into a significant reduction in hypoxia-induced factor HIF-1α levels. Further analyses revealed that immobilisation of the amorphous FeOOH shell on the UMOF support (UMOF@FeOOH) increased the degradation efficiency of H_2_O_2_ compared to free FeOOH, indicating an enhancement of catalase-like activity due to the coordination of Fe^3+^ ions with the carboxyl groups of UMOF. In vivo studies in a mouse model of subcutaneous HeLa xenografts confirmed the key role of FeOOH in the IMF system. Animals treated with photodynamic therapy using IMF and 808 nm laser irradiation showed a clear inhibition of tumour growth within the first two days after treatment, followed by a systematic reduction in tumour volume in the absence of further interventions. In the control group receiving IM nanoparticles (UMOF + ICG, devoid of FeOOH) and an identical irradiation regimen, no inhibition of tumour growth was observed, and tumour volume continued to increase. Moreover, two out of three mice in the IMF + laser group experienced complete tumour regression, providing promising evidence that overcoming hypoxia of the tumour microenvironment with FeOOH catalase-like activity can lead not only to inhibition of tumour growth but even to its complete eradication in a mouse model. Figure 2 shows the structure of IMF nanoparticles and their therapeutic mechanism [78].

## 3. Oxygen Generator

### Endoperoxides

Endoperoxides are compounds in which two oxygen atoms form a peroxide bridge (-O-O-) within a cyclic structure. They occur both as natural products and as synthetic compounds [79,80,81]. Cleavage of the -O-O- bridge leads to the release of molecular O_2_, which can modulate tissue hypoxia and improve cell survival in anoxic models [79]. Furthermore, some endoperoxides also exhibit anticancer properties through the production of reactive oxygen species [82,83]. Oxygen source endoperoxides were used in the work by Zhou et al. [84]. They developed M1-EPO-NPs as a triangular metallacycle with three aza-BODIPY units conjugated by acetylides to Pt(II) complexes. Two 1,4-dimethylanthracene groups were attached to each aza-BODIPY, storing singlet oxygen as endoperoxides and releasing it under heat. The whole was surrounded by DSPE-PEG2000 phospholipids. Compared to nanoparticles without endoperoxide groups, M1-EPO-NPs generated singlet oxygen under both normoxic (21% O_2_) and hypoxic (5% O_2_) conditions after laser irradiation, whereas for nanoparticles without endoperoxides, ^1^O_2_ production was only observed under normoxia. The increased efficiency of reactive oxygen species generation translated into stronger cytotoxicity against HeLa cells in vitro, with apoptosis rates of 71.8% in 21% O_2_ and 56.5% in 5% O_2_ for M1-EPO-NPs, compared to 62.6% (normoxia) and 44.3% (hypoxia) for endoperoxide-free nanoparticles. In a mouse model of HeLa tumours, M1-EPO-NPs nanoparticles led to complete tumour regression, whereas analogous formulations without endoperoxides failed to induce complete tumour eradication [84].

## 4. Inhibition of Mitochondrial Respiration

Mitochondrial respiration is the main process of oxygen consumption by living cells [85]. Although under hypoxia, tumour cells adaptively increase anaerobic glycolysis, the mitochondria still account for much of the ATP production by oxidative phosphorylation (OXPHOS) and consume most of the available O_2_ [86]. In view of this, strategies to pharmacologically inhibit the respiratory chain may temporarily reduce mitochondrial oxygen consumption, allowing oxygen to accumulate and be used more efficiently during PDT [87,88]. Two approaches have been used for this purpose in cervical cancer PDT: nitric oxide and atovaquone.

### 4.1. Nitrogen Monoxide (II)

Low levels of NO drive oncogenic pathways, immunosuppression, metastasis, and angiogenesis, while higher levels lead to apoptosis and reduced hypoxia, and sensitise tumour cells to conventional therapies [89]. The main NO derivatives, such as nitrogen dioxide and peroxynitrite, cause cell death by inducing protein and lipid peroxidation and/or DNA damage [90]. Importantly, excess NO can also affect mitochondrial respiration as it binds to cytochrome c oxidase, a critical component of the mitochondrial electron transport chain [91,92]. For this reason, NO generation may be an attractive form of increasing oxygen availability for PDT. A strategy based on the use of NO as a means of inhibiting mitochondrial respiration was used by Wang et al. [93] and Lin et al. [94] However, the source of NO differs in their work. To generate NO, Wang et al. used Roussin’s Black Salt [93]. Roussin’s Black Salt is a metal–nitrosine complex that releases NO upon illumination. Approximately 3.7 molecules of NO are released from one molecule of Roussin’s Black Salt. The relatively high cytotoxicity of Roussin’s Black Salt in the dark, however, precludes its use as a systemic anticancer agent in vivo, unless applied topically [95]. Designed by Wang et al. [93], the UM-RZ nanoparticle is composed of a core–shell–shell multilayer UCNP nanocrystal (NaErF_4_:Tm@NaYF_4_@NaYbF_4_:Tm@NaYF_4_), which converts near-infrared light into visible signals, asymmetrically deposited on a half-core shell of mesoporous silica, the photosensitiser zinc phthalocyanine, and Roussin’s Black Salt, described earlier. The mechanism of action of the UM-RZ nanoparticle relies on sequential activation. First, 980 nm light induces NO release, and then 808 nm light activates zinc phthalocyanine. In vitro studies conducted on HeLa cells demonstrated the high efficacy of using a nanoparticle containing Roussin’s Black Salt. Using only 808 nm radiation (ZnPc activation and ROS generation), a typical PDT-induced decrease in oxygen levels to ≈20% was observed, while earlier exposure of samples to 980 nm light (UM-RZ activation and NO release) resulted in inhibition of mitochondrial respiration, which maintained oxygen levels at a significantly higher level of ≈70%. In addition, NO generation from UM-RZ promoted active movement of nanoparticles and accelerated their internalisation by cells. Importantly, this approach also proved effective under hypoxia; while the use of the ‘808→980 nm’ sequence or simultaneous irradiation did not significantly improve the therapeutic effect, the ‘980→808 nm’ strategy (NO release first, followed by ROS generation) led to significant cytotoxicity under hypoxia (survival rate ≈ 15%), comparable to the effect observed in normoxia (≈9%), demonstrating an effective breakthrough of the hypoxia barrier. Results from in vivo studies on a mouse model of HeLa tumours confirmed that sequential activation of UM-RZ with 980 nm light, followed by 808 nm light, significantly increased the efficacy of PDT, leading to more than 86% inhibition of tumour growth [93].

For NO generation, Lin et al. used L-arginine [94]. L-arginine is a naturally occurring amino acid that is biocompatible, inexpensive, and produces a large number of NO molecules when catalysed by reactive oxygen species in PDT [96,97]. The UCN@mSiO_2_@ZnPc@L-Arg nanoparticle constructed by Lin et al. [94] consists of an upconverting NaYF_4_ core doped with Yb^3+^, Er^3+^, and Gd^3+^ ions, surrounded by two shells: NaLuF_4_:Y^3+^ and NaYF_4_. The whole was coated with mesoporous silicon oxide, allowing the loading of zinc phthalocyanine and L-arginine. In in vitro studies using HeLa cells, it was shown that the presence of L-arginine enables NO generation and modifies the kinetics of reactive oxygen species, as some oxygen radicals are consumed for the production of NO and nitro-oxygen derivatives. At the same time, it was confirmed that the level of intracellular ROS generation remains sufficient to induce a therapeutic effect. In the ONOO-specific fluorescence probe assay, only a weak signal was noted after irradiation with 980 nm light, suggesting the formation of small amounts of ONOO-. Furthermore, the presence of L-arginine was found to enhance the cytotoxic effects of PDT both in vitro and in vivo in a mouse model of HeLa tumours, compared to nanoparticles lacking L-arginine. However, it should be noted that the authors did not assess the effect of generated NO on mitochondrial respiration or oxygen bioavailability, which prevents a clear interpretation of the role of L-arginine as a strategy to counteract hypoxia [94].

### 4.2. Atovaquone

Atovaquone is a clinically approved antimalarial drug [98], which inhibits cytochrome bc_1_ function in the mitochondrial respiratory chain. By blocking oxidative phosphorylation [99], the main ATP production pathway in cancer cells [99], it alleviates tumour hypoxia and promotes the effectiveness of PDT [100,101]. Atovaquone was used in a nanoparticle by Gao et al. [102]. TNPs/IA is based on spherical nanostructures formed from mPEG-PLGA and PLGA-b-PEG copolymers modified with a triphenylphosphonium group. Two active compounds were encapsulated: IR780, a third-generation photosensitiser activated by NIR light (808 nm), and atovaquone. In vitro studies on HeLa cell lines showed that TNPs/IA effectively inhibited mitochondrial oxygen consumption. In TNPs/IA-treated cells, ATP levels decreased to only 37.8% of the initial value, providing convincing evidence for blocking the respiratory chain in mitochondria. Furthermore, under hypoxia, TNPs/IA generated the highest concentrations of reactive oxygen species relative to the control groups, as a result of the simultaneous inhibition of OXPHOS and the increased availability and precise localisation of IR780 in the mitochondria. The control groups lacking atovaquone (free IR780, NPs/I) and the group containing atovaquone, but without mitochondriotropic ligand (NPs/IA), showed significantly weaker effects both in vitro and in vivo on the mouse HeLa tumour model. This confirms that the synergistic combination of the OXPHOS inhibitor atovaquone and mitochondrial targeting is key to increasing oxygen availability in tumour cells and maximising the efficacy of PDT [102]. Table 2 shows summary of nanoparticles described in the article.

## 5. Conclusions

PDT is currently one of the promising minimally invasive treatments for cancer, including cervical cancer, due to its high selectivity of action and low systemic toxicity. However, its efficacy is often limited by hypoxia, commonly observed in solid tumours. To counter this phenomenon, over the past year, nanomedicine-based innovative strategies have been developed to increase oxygenation of the tumour microenvironment. Notable among these are catalytic oxygen generation from hydrogen peroxide, oxygen storage and controlled release, and inhibition of mitochondrial oxygen consumption in tumour cells. Preclinical studies have unequivocally demonstrated that all of these approaches result in an increase in oxygen concentration within the tumour and, consequently, significantly potentiate the efficacy of PDT. Importantly, the different approaches may have their own additional advantages, serving other functions, as well. MnO_2_-based nanomaterials, which act as a mesoporous carrier core, in addition to reversing hypoxia, increase the conversion of NIR radiation into heat, enhancing the photothermal effect, and release Mn^2+^ ions, initiating the Fenton reaction and generating hydroxyl radicals. Au_2_Pt hybrid systems exhibit peroxidase-like activity in the acidic environment of lysosomes, catalysing the formation of hydroxyl radicals (-OH) and thus further enhancing the efficacy of the therapy. The release of NO by Roussin’s Black Salt promotes the active transport of nanoparticles and accelerates their internalisation by cells, while NO generated from L-arginine initiates the formation of highly reactive ONOO- radicals. Moreover, the integration of these solutions in multifunctional platforms combining photodynamic, photothermal, and chemodynamic mechanisms allows not only to overcome the multidimensional barriers of cellular resistance but also to precisely target subsequent therapeutic agents while minimising damage to healthy tissues. Despite promising results from in vitro and in vivo studies, further rigorous safety, biocompatibility, and efficacy testing in preclinical models remains crucial to fully confirm the suitability of these nanosystems in clinical practice. In light of the accumulated evidence, nanocarriers that modulate oxygen levels in the tumour microenvironment provide a solid foundation for future, more selective and individualised photodynamic treatment strategies for cervical cancer, with the prospect of extending their application to other solid tumours.

## 6. Recommendations

Based on our analysis, we recommend that further work on nanomaterials for hypoxia modulation in photodynamic cancer therapy should focus primarily on the development of catalytic platforms that degrade H_2_O_2_ (e.g., MnO_2_, FeOOH) and on oxygen storage and controlled release systems (endoperoxides), which have shown the most consistent improvement in oxygenation and proven therapeutic benefit in preclinical studies. In parallel, we advocate the design of multifunctional systems combining local oxygen production with mechanisms that limit oxygen consumption by tumour cells (e.g., encapsulation of OXPHOS inhibitors or controlled release of NO), as data show synergy of these approaches in enhancing ROS generation and PDT efficacy. To facilitate comparability of results and expedite the identification of candidates for clinical translation, we recommend a minimum reporting standard including pO_2_ pre- and post-therapy measurements, quantification of HIF-1α expression, quantification of ROS, measures of efficacy (e.g., % inhibition or tumor regression), and complete biodistribution and toxicology data. In addition, we recommend direct head-to-head comparisons of the most promising constructs in the same models and protocols, and expanded pharmacokinetic studies and long-term safety assessments (metal ion fate, material degradation, immunotoxicity). Finally, it is advisable to study the optimal sequence and timing of activation of multifunctional components (e.g., staged release of NO or inhibition of OXPHOS prior to activation of the photosensitiser), since the sequence of events adopted has a significant impact on the oxygenation of the microenvironment and the therapeutic outcome.

## Figures and Tables

**Table 1:** Criteria for inclusion and exclusion of retrieved articles for overgrowth.

Inclusion Criteria
Articles describing photodynamic therapy
Articles describing cancer therapy
Articles describing nanoparticles
Articles published in 2024 and by March 2025
**Exclusion Criteria**
Articles describing nanoparticles without a strategy for overcoming hypoxia
Articles describing cancers other than cervical cancer
Articles other than original research papers
Articles in which the results of therapy were described only in vitro
Articles in a language other than English and Polish

**Table 2:** Summary of nanoparticles described in the article.

Nanoparticle	Construction	Hypoxia Reversal Method	Effectiveness of Hypoxia Reversal	Therapeutic Efficacy	Reference
DMPIM	Mesoporous silica + MnO_2_ + green indocyanine + doxorubicin + polydopamine	Decomposition of H_2_O_2_ to O_2_ via MnO_2_	Increase in ROS production relative to NP without MnO_2_	92.91% inhibition of tumour growth in a mouse model of U14	[68]
DPIMGC	Doxorubicin + polidopamine + green indocyanine + MnO_2_ + gelatin with celecoxib	Decomposition of H_2_O_2_ to O_2_ via MnO_2_	Increased ROS production relative to NP without MnO_2_, decreased HIF-1α in musim model U14	91.47% inhibition of tumour growth in a mouse model of U14	[67]
M-HMnO_2_@ICG	MnO_2_ core + poly(allylamine hydrochloride) + green indocyanine + HeLa cell membrane	Distribution of H_2_O_2_ to O_2_ via MnO_2_	Downregulation of HIF-1α in a mouse model of HeLa	88% inhibition of tumour growth in a mouse model of HeLa	[70]
LRZAPH	LuAG:Tb/Ce + Rose Bengal + ZIF-8 + Au_2_Pt + hyaluronic acid	Catalase/peroxidase activity Au_2_Pt	Increase in ROS production relative to NP without Au_2_Pt ^1^	93% inhibition of tumour growth in a mouse model of HeLa	[73]
IMF (ICG ⊂ UMOF@FeOOH)	UMOF + FeOOH + ICG coating	Decomposition of H_2_O_2_ to O_2_ via FeOOH	Downregulation of HIF-1α in normoxia and hypoxia	Complete regression of 2/3 of tumours in a mouse model of HeLa	[78]
M1-EPO-NPs	Metallacycle + aza-BODIPY + endoperoxides + DSPE-PEG2000	Release of O_2_ from endoperoxides	Generation of singlet oxygen in both normoxia and hypoxia by M1-EPO-NPs relative to NPs without endoperoxides, in which ^1^O_2_ production occurred exclusively in normoxia	Complete tumour regression in a mouse model of HeLa	[84]
UM-RZ	UCNP + mesoporous silica + ZnPc + Roussin’s Black Salt	NO-mediated inhibition of mitochondrial respiration	Activation of ZnPc with 808 nm light led to a decrease in oxygen levels to ≈20%, while pre-exposure to 980 nm (UM-RZ activation and NO release) maintained oxygen levels at ≈70%.	86% tumour inhibition in a mouse model of HeLa	[93]
UCN@mSiO_2_@ZnPc@L-Arg	UCNP + mesoporous silica + ZnPc + L-arginine	NO-mediated inhibition of mitochondrial respiration	No clear assessment of hypoxia	78.5% inhibition of tumour growth in a mouse model of HeLa	[94]
TNPs/IA	mPEG-PLGA + PLGA-b-PEG + IR780 + atovaquone + TPP^+^	OXPHOS inhibition (atovaquone)	Increased ROS production under hypoxia relative to NP without atovaquone	Highest in vivo efficacy in a mouse model of HeLa relative to control groups	[102]

^1^ Au_2_Pt exhibits catalase activity over a wide pH range from weakly acidic to basic, with maximum efficiency at pH ≈ 8, while under low pH conditions, peroxidase-like reactions leading to the generation of -OH radicals dominate, at the expense of catalase activity [73].
